# Investigating Research Hotspots and Publication Trends of Spinal Stenosis: A Bibliometric Analysis During 2000–2018

**DOI:** 10.3389/fmed.2021.556022

**Published:** 2021-07-20

**Authors:** Keda Yang, Lei Pei, Kaicheng Wen, Siming Zhou, Lin Tao

**Affiliations:** Department of Orthopaedics, First Hospital of China Medical University, Shenyang, China

**Keywords:** research hotspots, publication trends, spinal stenosis, bibliometric analysis, MeSH terms

## Abstract

Spinal stenosis is a common disease affecting the elderly that is present in a various forms. Its high incidence forces researchers to pay more attention and offer countermeasures. We used the Web of Science Core collection and PubMed database to obtain 5,606 scientific studies concerning spinal stenosis, and the number of publications maintained a roughly increasing trend from 108 in 2000 to 512 in 2018, only declining in 2011. Bibliometric analysis was conducted using the online analysis software CiteSpace and Bibliographic Item Co-Occurrence Matrix Builder (BICOMB). The United States maintains academic leadership in this field. The journal SPINE was the most authoritative, with 695 articles and an average of 12.73 citations. The exported major MeSH terms were further biclustered with gCLUTO according to co-word analysis to reveal research hotspots, including etiology, pathogenesis, clinical manifestation, conservative treatment, operative indication, internal implantation, and postoperative complications. After combination, the main topics focused on pathogenesis and surgical treatment. Narrowing causes flavum ligamentum hypertrophy, and posterior longitudinal ligament ossification is widely accepted. Additionally, minimally invasive surgery and internal implantation fixation are more valid in the clinic. Refining pathological classification and optimizing surgical methods and instrument properties will be important future research directions for spinal stenosis.

## Introduction

Spinal stenosis involves pathological symptoms, such as narrowing of the spinal canal and shortening of the canal diameters, compression of the spinal cord, and the presence of neurological disturbance. According to the vertebral segments, narrowing occurs commonly in the cervical and lumbar spine and rarely has a thoracic appearance. An increasing risk of limb dysfunction and urinary and fecal incontinence exists in elderly individuals with spinal stenosis. By dissecting the anatomical structure of the spinal canal, bone hyperplasia and fibrous tissue adaptation are the main risks of spinal stenosis (1, 2). Additionally, the local inflammatory microenvironment, calcium maladjustment, and microbial infection contribute to disease development (3). Measurement of the canal diameter, observation of symptoms, and auxiliary examination with radiologic technology is the gold standard for a definite diagnosis and is widely adopted in the clinic. Once diagnosed, treatment varies according to the narrowing degree and clinical manifestation. Mild stenosis is usually asymptomatic and difficult to detect. No intervention is available that does not affect the normal life quality of life. Non-operation therapy is the first choice, including functional exercise, massage, and traction. The application of drugs, blockade, and acupuncture remains relatively controversial. However, if nervous compression manifestations, such as pain, numbness, and dysfunction of physical activity, have seriously affected severe stenosis patients (4), the treatment process is usually complicated, particularly in elderly individuals with vascular and neurological claudication. Clinical studies have shown that, among patients with lumbar spinal stenosis, with or without degenerative spondylolisthesis, decompression surgery plus fusion surgery did not result in better clinical outcomes at 2 and 5 years than decompression surgery alone (5). However, posterolateral instrumented fusion showed a greater improvement in the quality of life than decompression alone in another comparison (6). As discussed above, controversies exist regarding surgical choice and postoperative efficacy. Thus, the field of spinal stenosis warrants further exploration.

Bibliometrics, a cross-science of quantitative analysis of all knowledge carriers using mathematical and statistical methods, evaluates scientific achievements and treatises and predicts research trends in a particular field. It is a comprehensive knowledge system that integrates mathematics, statistics, and bibliography, mainly focusing on quantification. The number of documents, authors, and vocabularies are the measurement objects. Thus, an essential feature of bibliometrics is that the output must be “quantity.” Quantitative research on the literature can be traced back to the beginning of the 20th century. In 1969, Alan Pritchard named this quantitative analysis method “bibliometrics.” Recently, an increasing number of studies have assessed the contribution and trends of publications in various areas. Yao et al. regarded immunosuppression as a hotspot of sepsis by clustering keywords (7). Ahmad et al. identified the high-risk factors of Periodontology 2000 by synthesizing the study results of extensive dental institutions (8). These studies supported the reliability of bibliometrics in evaluating hotspots and directing trends of scientific research.

Presently, knowledge updates are constantly accelerating, and the experience of previous experts is slightly lagging, creating challenges for medical workers. Bibliometrics can effectively improve this situation. Before this study, no bibliometric articles were available on spinal stenosis. We adopted the biclustering method of bibliometrics to provide a comprehensive analysis of the current status of spinal stenosis. Our study aims to uncover hotspots and predict trends based on bibliometric analysis.

## Methods

### Data Source and Collection

All literature was retrieved from the Web of Science Core Collection (WoS) from January 2000 to December 2018. The retrieving process was completed in Dec 30, 2019 to reduce the deviation within a constantly updated database. The key indexes of our study were “spinal stenosis” and “English” for language. Five thousand six hundred and six pieces of literature were collected and the document types included ARTICLE and REVIEW, improving the comprehensive feature of our research. Medical subjects Headings (MeSH) terms express the main idea of a piece of literature, identified for the following co-word associated analysis (9). For document download, we used the PubMed database, a biomedical information retrieval system developed by National Center for Biotechnology Information (NCBI) that was relatively complete in medicine. It was convenient to use the MeSH terms directly on PubMed as the keywords with a higher relevance. Our data obtained from the Web of Science Core Collection was converted into txt format first and conducted for quantitative and statistical analysis by CiteSpace, a “citation space” for visualization and the Online Analysis Platform of Literature Metrology (http://bibliometric.com/) for bibliometrics. The downloaded documents were also converted into txt format and inducted the research generality and evolutionary trend according to co-word and biclustering analysis of MeSH terms by Bibliographic Item Co-Occurrence Matrix Builder (BICOMB) and gCLUTO. It was necessary to note that three investigators carried out literature screening independently based on the abstracts, conclusions, or full articles in our preliminary experiment. The consistency was up to 0.95. And we discussed the differences and reached the consensus, which reflected the reliability of our data retrieval.

### Data Analysis

We divided the analysis process into three parts: content association, high-frequency MeSH terms filtering, and scientific direction prediction.

Citespace is an excellent bibliometric software that can reveal the potential connection between documents visually with the form of scientific knowledge maps (10). It is developed based on the WoS data format. Cooperative network analysis, co-occurrence analysis, and co-citation analysis can be performed according to the downloaded data. But data from non-WoS databases need to be converted to the WoS data format first. And data dimensions of the corresponding database applies to its corresponding scope. We concatenated the retrieved publications with the similarity in journals, publication years, countries, authors, and languages by using Citespace, which helped us select nodes for different focuses conveniently and precisely.

BICOMB were developed by Cui et al. from Department of Information Management and Information System (Medical) in China Medical University (11). Major MeSH terms could be exported to represent the publication core and rank the occurring frequency with Microsoft Excel and GoPubMed. The screening publications were analyzed and checked with a designed model in XML format to extract the main information in the beginning. The filter of high-frequency MeSH terms was performed in a binary matrix after preliminary interception through a particular threshold. And the latent biomedical principle could be excavated with association and clustering analysis in terms of the results from a binary matrix. Then both the major MeSH terms and their networks were conducted for visualization in graph.

gCLUTO were utilized to carry out biclustering analysis of the major MeSH terms from BICOMB. There are four cluster mothed in gCLUTO—Repeated Bisection, Direct, Agglomerative, and Graph—among which we selected Repeated Bisection with the highest preciseness. And the classified results of these terms would be extracted in two forms: matrix visualization and mountain visualization (12). In matrix visualization, colors represented values in the raw data matrix. White equaled the central value “zero,” positive values increased with red deepening, and negative values were depicted with green. Rows of matrix held columns with the same class. Mountain visualization could make accurate predictions with multidimensional scaling. Peak number reflected the clustering number in matrix. Mountain shape provided a rough estimate of data distribution in each cluster. The peak altitude is proportional to the similarity and volume is proportional to the number of containing objects in a cluster. Additionally, the peak color is related to the standard deviation within the cluster: red for low value and blue for high value. And we measured the clustering number constantly to reach the optimal visualized graph to investigate the research hotspots and publication trends of spinal stenosis.

## Results

### Quantity of Relevant Literature

**Figure 1:**
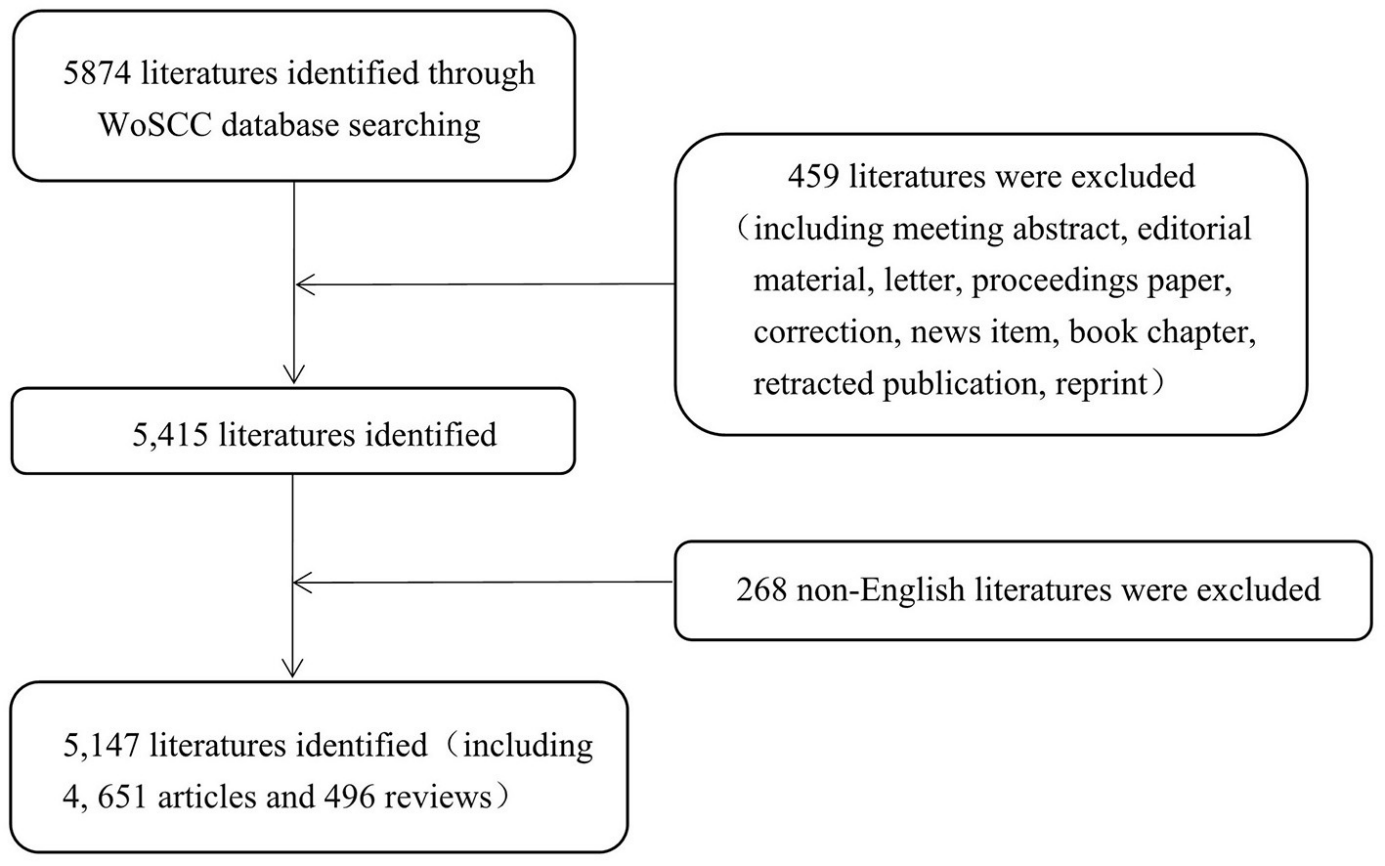
Flow chart of literature filtering involved in this study.

**Figure 2:**
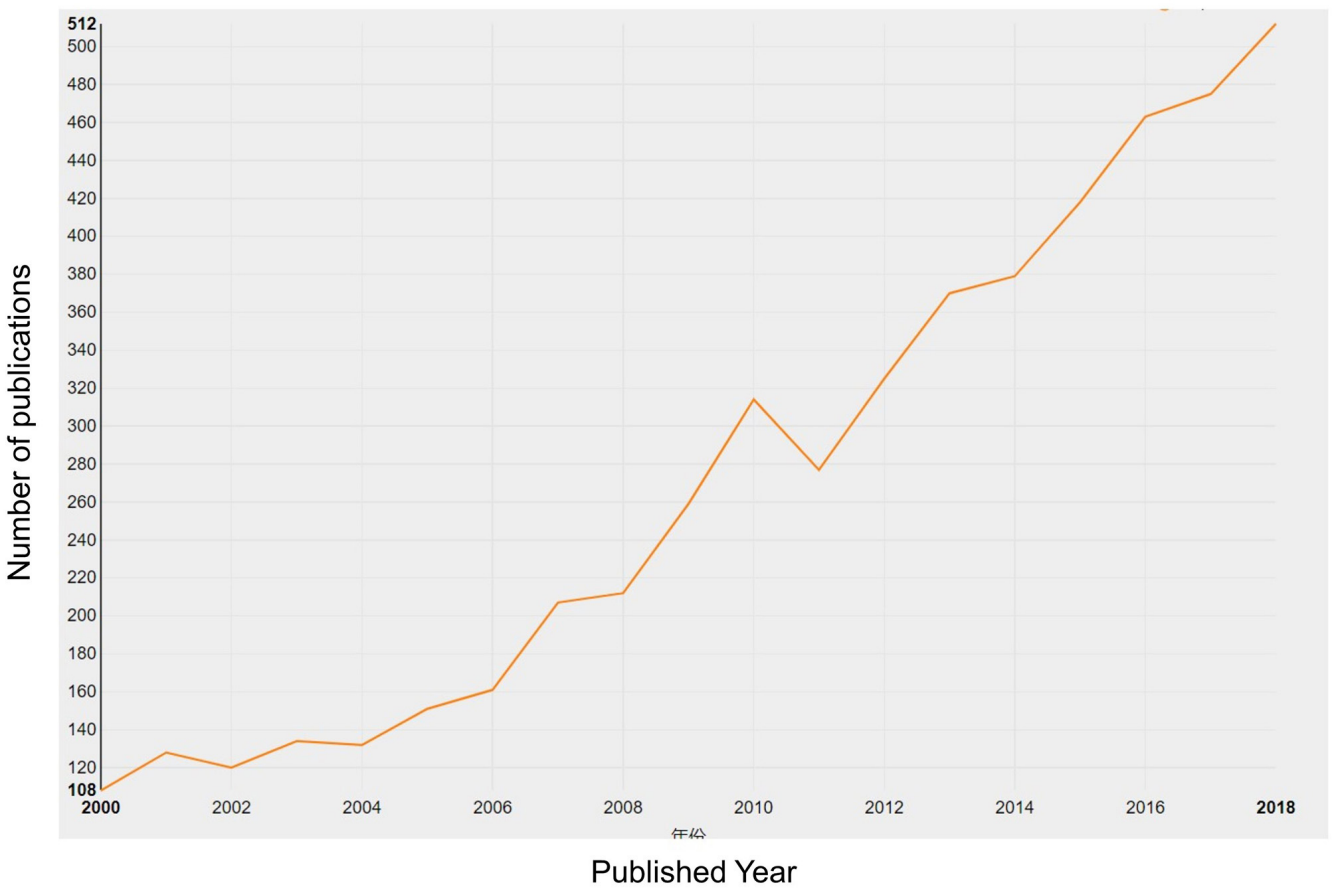
Quantity of relevant literature. The number of annual publications in spinal stenosis from 2000 to 2018.

Regarding literature screening, we set up the keywords as “spinal stenosis” and “language = English.” Our retrieval period ranged from January 1, 2000, to December 31, 2018. In total, 5,147 publications were involved in 4,651 articles and 496 reviews (Figure 1). The number of publications generally increased from 108 in 2000 to 512 in 2018, and only declined in 2011 (Figure 2). Additionally, the growth rate was also improving.

### Distribution Characteristics of Literature

#### Most Active Countries or Regions

**Figure 3:**
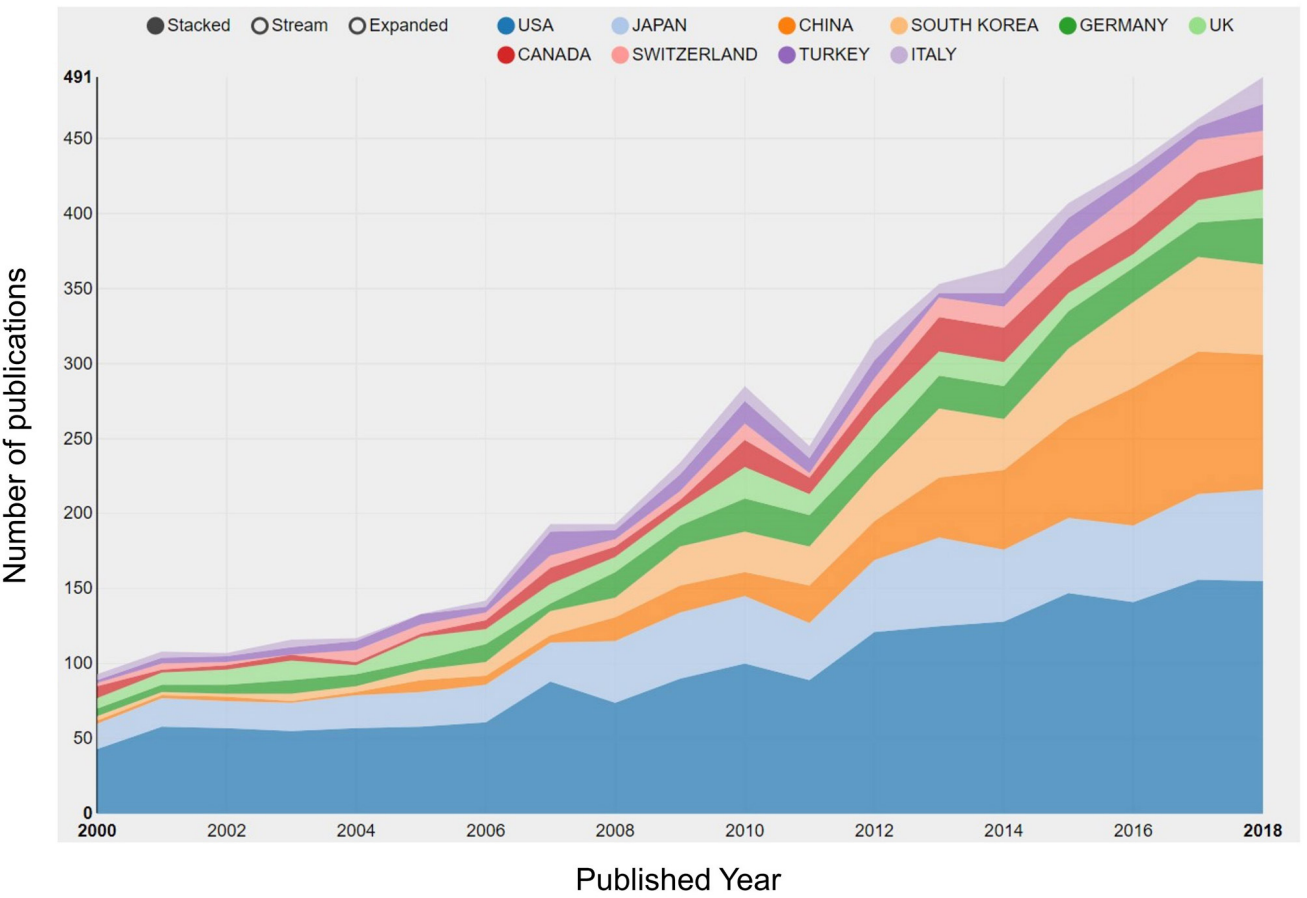
Quantity of relevant literature. The development of the top 10 countries/regions in spinal stenosis from 2000 to 2018.

**Figure 4:**
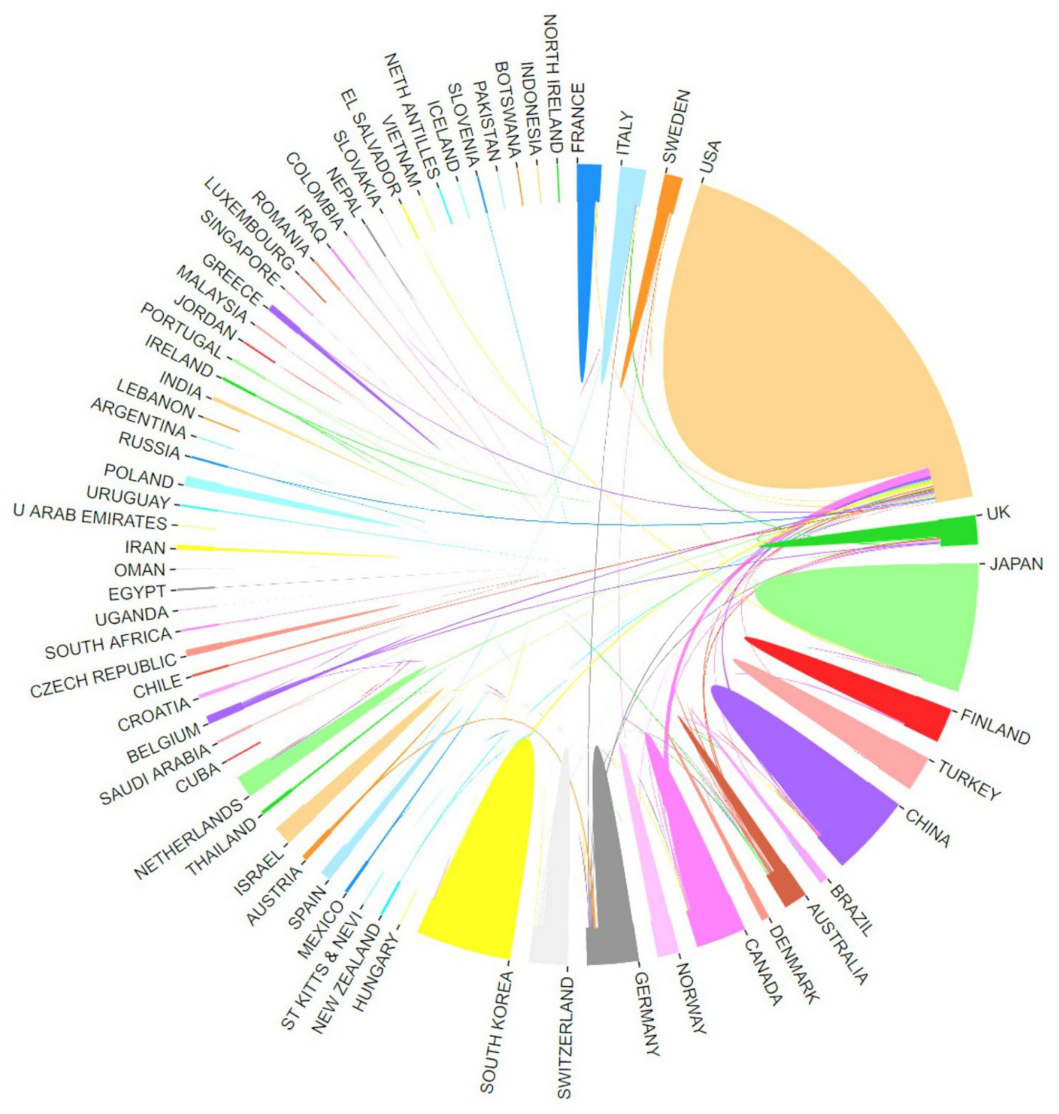
Most active countries or regions. The cooperation of countries/regions involved in spinal stenosis research.

The county or regional distribution of publications in bibliometric analysis has focused on the hotspots of research in spinal stenosis by refining data sources. In our study, we demonstrated the top ten countries leading in the field of spinal stenosis according to the publication counts (Figure 3). The United States was far ahead of other counties with 1,803 studies, followed by Japan (711), China (566), and South Korea (479). Notably, the research level in Northeast Asian countries had gradually increased and surpassed that in Europe over time. We attributed this phenomenon to economic development. Additionally, the centrality index also reflected the core influence to evaluate research value more exactly. The United States maintained a leading position with 0.71 centrality. All of the other countries had a lower influence, among which Germany had the highest centrality at 0.23 (Table 1). The cooperative network was formed between many countries and the level was relatively higher in leading countries (Figure 4).

**Table 1:** The top 10 countries/regions: article counts and centrality of publications in spinal stenosis research.

**Rank**	**Country/region**	**Article counts**	**Centrality index**
1	USA	1,803	0.71
2	Japan	711	0.03
3	China	566	0.01
4	South Korea	479	0.01
5	Germany	293	0.23
6	UK	248	0.14
7	Canada	218	0.08
8	Switzerland	173	0.04
9	Turkey	169	0.01
10	Italy	131	0.01

#### Top Ten Active Institutions

**Figure 5:**
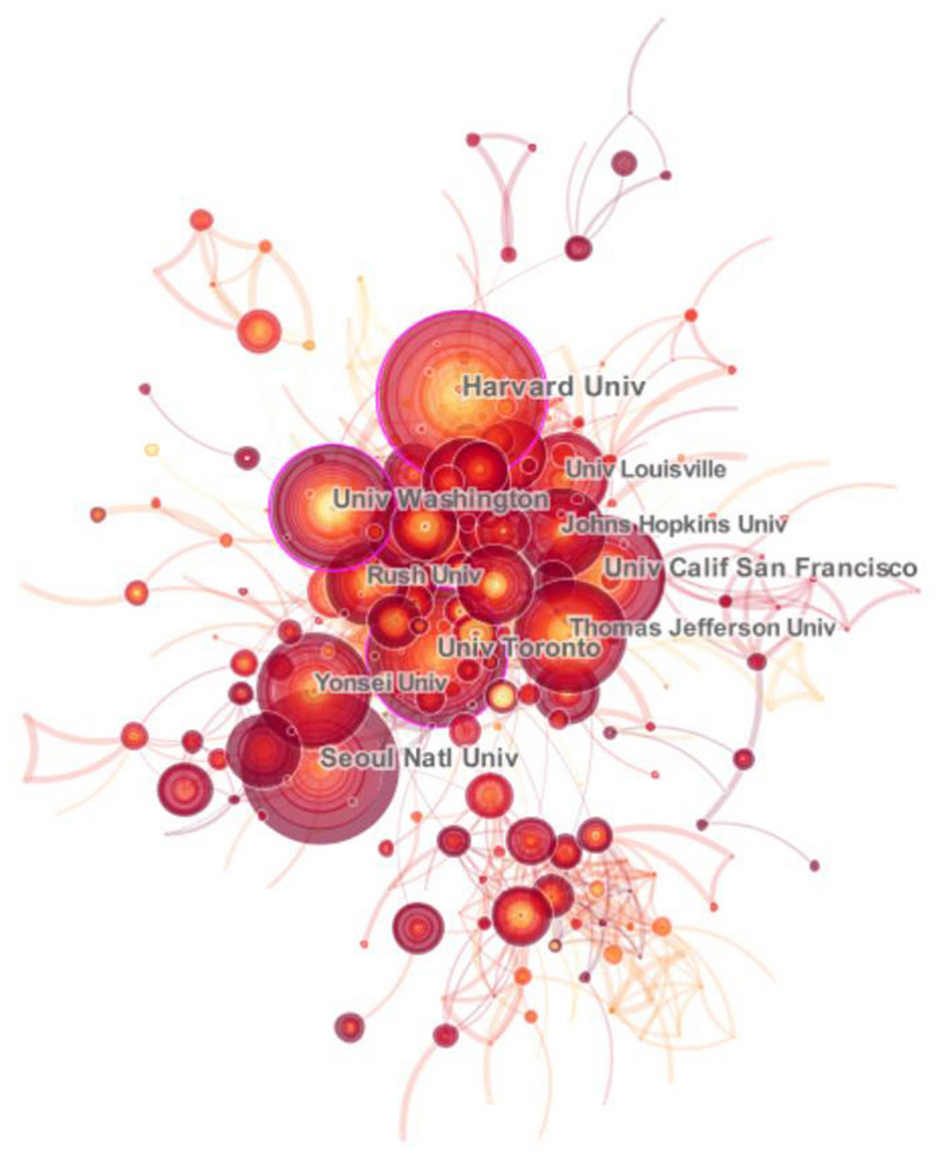
Top ten active institutions. The collaborative network of institutions involved in spinal stenosis research.

We also demonstrated the top ten institutions for spinal stenosis research ordered by the publication number. They were Seoul Natl Univ, Univ Washington, Univ Toronto, Harvard Univ, Yonsei Univ, Kuopio Univ Hosp, Cleveland Clin, Univ Calif San Francisco, Vanderbilt Univ, and Johns Hopkins Univ (Table 2). Among them, more than half were from the United States, two institutions were from Korea, and only one belonged to Finland and Canada, respectively, in line with the distribution characteristics of countries. Seoul Natl Univ (231), Univ Washington (169), Univ Toronto (155), and Harvard Univ (127) published more than one hundred articles and their average citations were 3.14, 20.17, 6.14, and 23.63, respectively. It was clear to see that the institutions in the United States had relatively higher average citation and centrality, which determined the credibility (Table 2). With extensive academic communication among scholars, it was necessary to develop closer research collaboration between various institutions for the lower level (0.015 on density map) (Figure 5).

**Table 2:** The top 10 institutions for most publications in spinal stenosis research.

**Rank**	**Institution**	**Article** **counts**	**Total** **number of** **citations**	**Average** **number of** **citations**	**Total number** **of first** **authors**	**Total number** **of first** **author citations**	**Average number** **of first** **author citations**	**Centrality** **index**	**Country**
1	Seoul Natl Univ	231	725	3.14	64	259	4.05	0.04	Korea
2	Univ Washington	169	3,408	20.17	33	369	11.18	0.1	US
3	Univ Toronto	155	994	6.41	31	157	5.06	0.12	Canada
4	Harvard Univ	127	3,001	23.63	34	606	17.82	0.15	US
5	Yonsei Univ	97	363	3.74	41	157	3.83	0.03	Korea
6	Kuopio Univ Hosp	95	1,543	16.24	23	194	8.43		Finland
7	Cleveland Clin	88	386	4.39	25	99	3.96	0.02	US
8	Univ Calif San Francisco	87	1,344	15.45	35	128	3.66	0.1	US
9	Vanderbilt Univ	86	1,815	21.1	32	161	5.03	0.03	US
10	Johns Hopkins Univ	84	194	2.31	34	62	1.82	0.02	US

#### Distribution Characteristics of Authors and Journals

**Figure 6:**
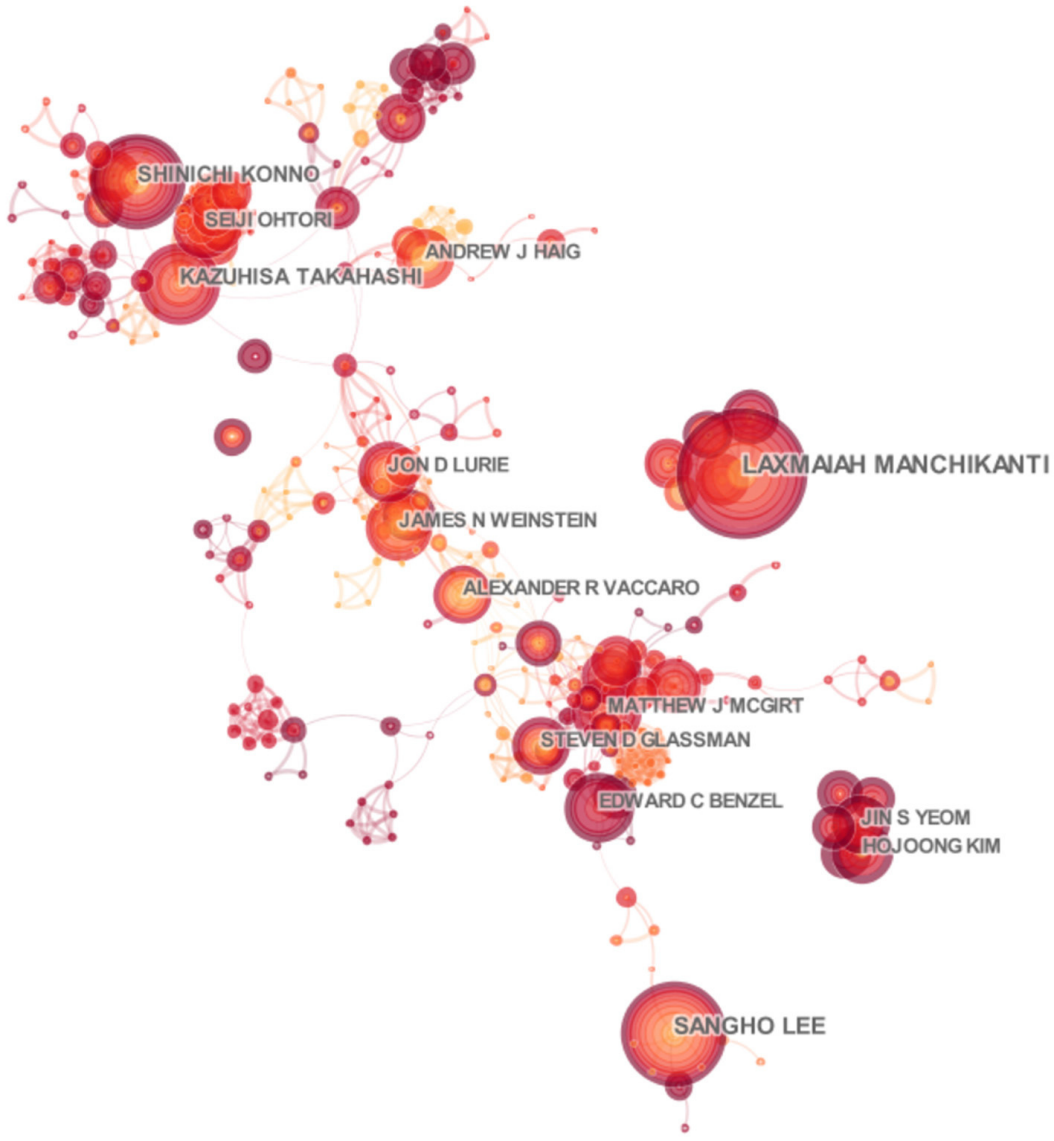
Distribution characteristics of authors. The associated network of productive authors.

**Figure 7:**
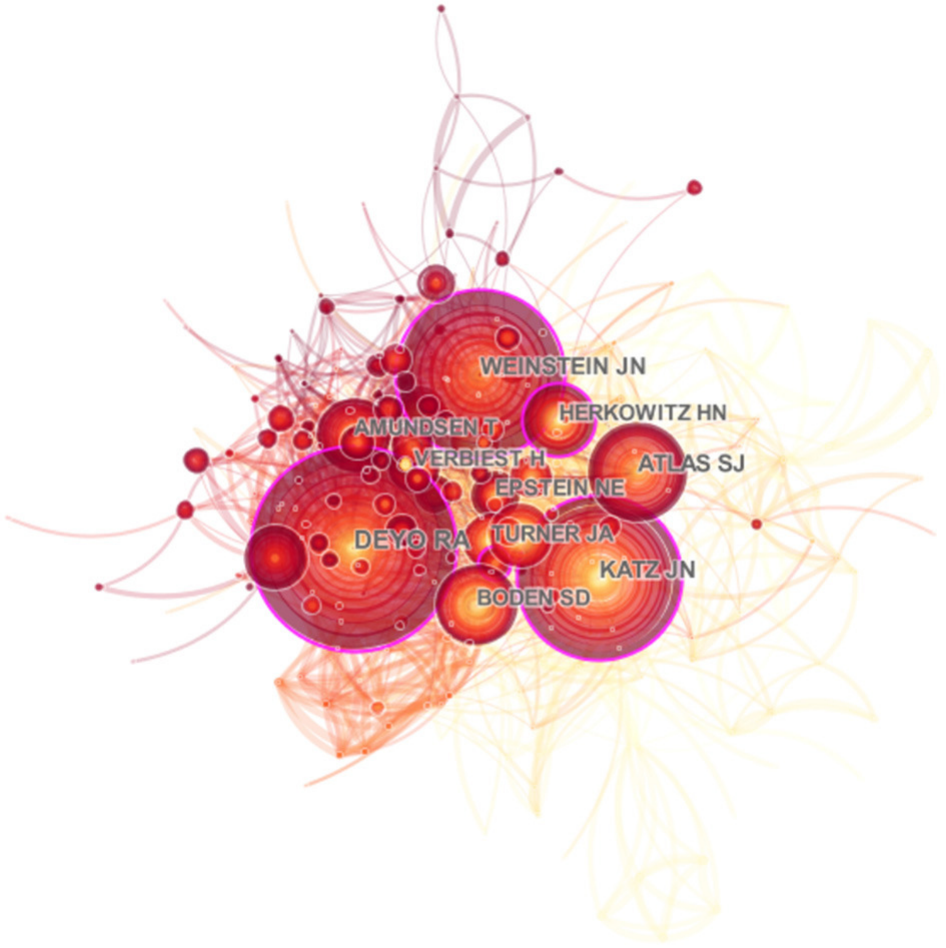
Distribution characteristics of authors. The associated network of co-cited authors.

There were a total of 15,718 authors involved in the field of spinal stenosis through our retrieval. We paid more attention to the ten most active authors including Lee SH, Kim HJ, Manchikanti L, Lee JH, and Konno S in order of published counts (Table 3). The top three among them were Lee SH, Kim HJ, and Manchikanti L with the number of 73, 60, and 54. They came from Pusan National University Yangsan Hospital in Korea, Seoul National University Bundang Hospital in Korea, and Pain Management Center of Paducah in America, respectively. It was noted that they contributed most to the development of the scientific study, though there was not a large gap with other authors, which also showed the research momentum of spinal stenosis. We drew an associated network based on the integration of cited and co-cited authors by CiteSpace at the same time (Figures 6, 7). Deyo RA was the co-cited author with the most co-citation (812). Katz JN (659) and Weinstein JN (630), close to each other, ranked second and third. Additionally, the centrality index of them was 0.18, 0.10, and 0.10 which reflected the credibility and authority of their research (Table 3).

**Table 3:** The top 10 most productive authors and co-cited authors contributing to publications in spinal stenosis research.

**Rank**	**Author**	**Article** **counts**	**Total number** **of citations**	**First author** **citation counts**	**Corresponding** **author**	**Co-cited** **author**	**Citation** **counts**	**Centrality** **index**
1	Lee, SH	73	360	37	10	Deyo RA	812	0.18
2	Kim, HJ	60	269	126	3	Katz JN	659	0.1
3	Manchikanti, L	54	1,610	1,520	50	Weinstein JN	630	0.1
4	Lee, JH	46	163	103	16	Atlas SJ	448	0.09
5	Konno, S	45	294	93	3	Boden SD	397	0.08
6	Kikuchi, S	39	317	0	0	Amundsen T	383	0.1
7	Lurie, JD	37	1,290	113	9	Herkowitz HN	347	0.16
8	Weinstein, JN	36	1,431	778	7	Verbiest H	309	0.08
9	Watanabe, K	36	176	61	7	Turner JA	289	0.07
10	Vaccaro, AR	34	234	50	11	Epstein NE	288	0.08

In total, there were 735 journals involved in this field. SPINE and EUROPEAN SPINE JOURNAL are the top two journals in order of article counts and total number of citations which were 695, 8,847, and 426, 2,908, respectively. Both of them were Q2 in JCR 2018 standards. The other journals in the top ten journals were shown in Table 4. The average number of citations and the impact factor were also important indicators for evaluating the influence of journals. PAIN PHYSICIAN (15.91) was the highest in terms of the average number of citations, followed by SPINE (12.73) and JOURNAL OF NEUROSURGERY-SPINE (7.09). According to the impact factor, JOURNAL OF SPINE JOURNAL (3.196) was the highest, JOURNAL OF NEUROSURGERY-SPINE (2.998) ranked second, and PAIN PHYSICIAN (2.942) ranked third. The number of articles published in these ten journals accounted for 42.47%. It was obvious that these ten journals laid a solid foundation for subsequent research on spinal stenosis.

**Table 4:** The top 10 journals contributing to publish articles in spinal stenosis research.

**Rank**	**Institution**	**Article** **counts**	**Percentage** **(*N*/5,147)**	**Total number** **of citations**	**Average number** **of citations**	**IF** **(2018)**	**Quartile in** **category (2018)**	**H-index**
1	Spine	695	13.50%	8,847	12.73	2.903	Q2	228
2	European Spine Journal	426	8.28%	2,908	6.83	2.513	Q2	117
3	Spine Journal	221	4.29%	1,148	5.19	3.196	Q0	94
4	Journal of Neurosurgery-Spine	215	4.18%	1,525	7.09	2.998	Q3	84
5	World Neurosurgery	148	2.88%	131	0.89	1.723	Q0	85
6	Journal of Spinal Disorders & Techniques	133	2.58%	936	7.04	0	Q4	85
7	Pain Physician	123	2.39%	1,957	15.91	2.942	Q0	87
8	Neurosurgery	88	1.71%	557	6.33	4.13	Q3	183
9	BMC Musculoskeletal Disorders	69	1.34%	350	5.07	2.002	Q3	81
10	Journal of Korean Neurosurgical Society	68	1.32%	123	1.81	1.187	Q0	29

#### Research Hotspots of Spinal Stenosis Based on MeSH Clusters

**Figure 8:**
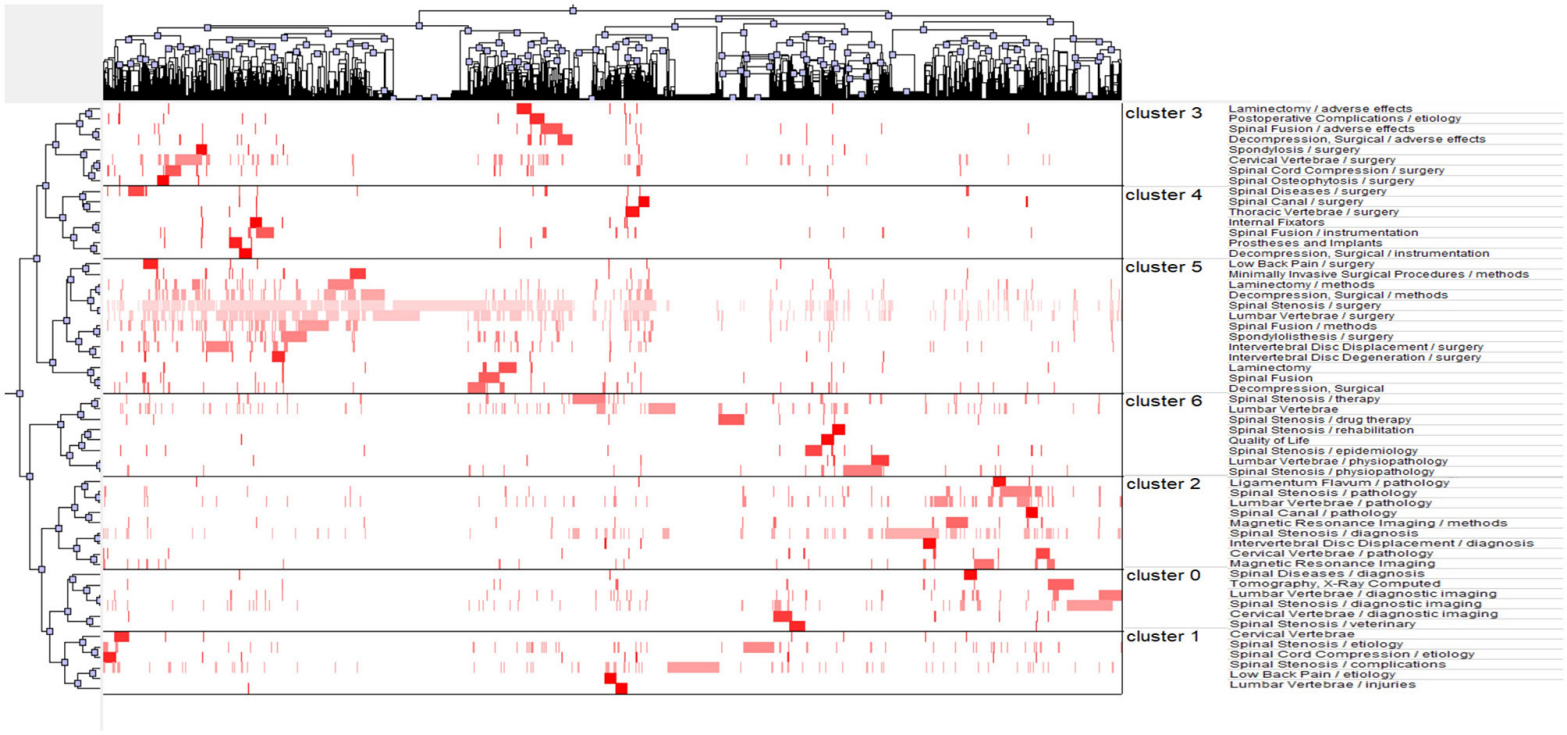
Matrix visualization of biclustering of high-frequency major MeSH terms and PMIDs of articles on spinal stenosis.

**Figure 9:**
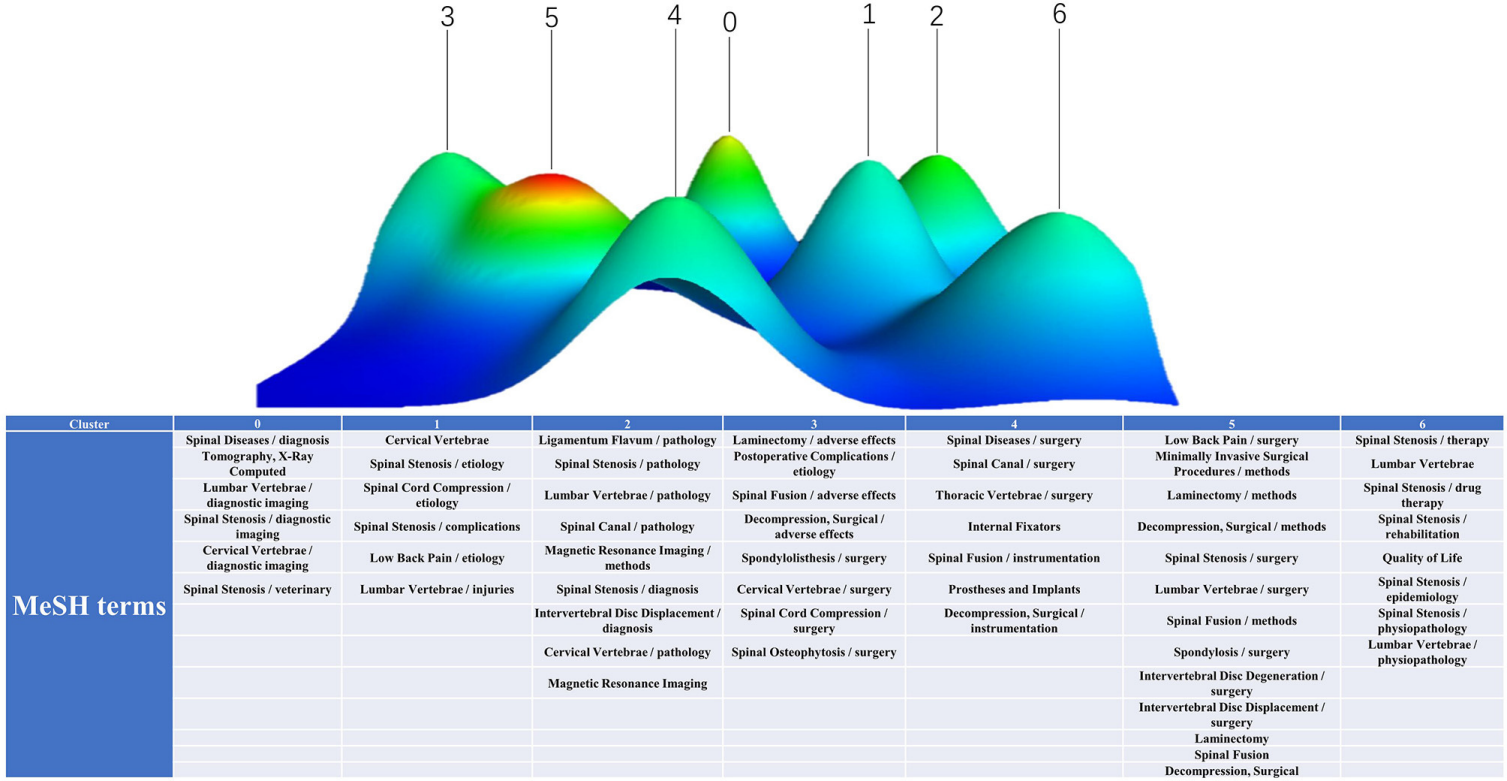
Mountain visualization of biclustering of high-frequency major MeSH terms and articles on spinal stenosis.

Through statistical and quantitative analyses of the publications from January 1, 2000, to December 31, 2018, 3,810 major MeSH and MeSH subheading terms were identified, with a total frequency of 23,785 times. After repeatedly proofreading and balancing, the major MeSH and MeSH subheading terms with occurrence frequencies of 56 times and above were regarded as high-frequency terms. Altogether, fifty-seven terms were high in appearance with a total frequency of 11,754, accounting for 49.42% (11,754/23,785) (Table 5). The major MeSH terms/subheading MeSH terms, spinal stenosis/surgery and lumbar vertebrae/surgery, appeared more than one thousand or even close to two thousand times, reflecting the predilection segment of lumbar vertebrae and the treatment method of surgery. Biclustering helped classify these high-frequency major MeSH and MeSH subheading terms, convenient to refine the main topics of spinal stenosis. Thereafter, the results were visualized in the form of a matrix and mountain graph. The color was the core property to reflect the raw matrix value in matrix visualization. The balanced point was white; red represented a positive value, and green represented a negative value, both of which expressed a positive correlation. In the matrix graph, clusters are separated by black horizontal lines, and each row is an individual category. Thus, seven clusters were observed in our matrix (Figure 8). The row and column tags represented the high-frequency major MeSH terms/MeSH subheading terms and the PMIDs of articles, respectively. When the matrix was exhibited in the dendrogram, the top and left layers revealed the association with different indexes. In Figure 8, the top tree indicates the relationships among articles, and the left tree indicates the relationships among high-frequency major MeSH terms/MeSH subheading terms. gCLUTO could also rearrange the attitude of rows in the initial matrix to integrate similar rows (Table 6). In our mountain visualization, seven peaks existed from 0 to 6, revealing seven clusters after biclustering (Figure 9). In the 3D landform, the properties of each cluster were visualized by comparing the location, volume, altitude, and color of the corresponding peaks. The location of peaks represents the relative similarity among different clusters, with a higher location for closer clusters. Both the volume and altitude referred to the internal features in clusters. A greater volume reflected more objects containing major MeSH and MeSH subheading terms, and the altitude demonstrated the internal similarity in direct proportion. Furthermore, the color of the peak reflected the deviation degree of the dataset, equal to the standard deviation. Cluster 5 with a relative red peak referred to a lower value and a higher value with the trend of bluing in our study (Figure 9). Similar to our analysis above, the major MeSH and MeSH subheading terms were refined into the following seven clusters:

Effect of medical imaging on the diagnosis of spinal stenosis (Cluster 0),
Etiology of spinal stenosis with different pathological symptoms (Cluster 1),
Pathogenesis of spinal stenosis (Cluster 2),
Options of surgical method under particular conditions and adverse effects (Cluster 3),
Role of instrumentations in rehabilitation of patients (Cluster 4),
Surgical indications of spinal stenosis (Cluster 5),
Non-operative therapeutic means of spinal stenosis (Cluster 6).

**Table 5:** High-frequency major MeSH terms from the involved publications on spinal stenosis (*n* = 23,785).

**Rank**	**Major MeSH terms/ MeSH subheadings**	**Frequency**	**Proportion of frequency (%)**	**Cumulative percentage (%)**
1	Spinal stenosis/surgery	1,998	8.4003	8.4003
2	Lumbar vertebrae/surgery	1,182	4.9695	13.3698
3	Spinal stenosis/diagnosis	535	2.2493	15.6191
4	Decompression, surgical/methods	472	1.9844	17.6035
5	Spinal stenosis/complications	397	1.6691	19.2727
6	Spinal fusion/methods	377	1.5850	20.8577
7	Spinal stenosis/diagnostic imaging	361	1.5178	22.3754
8	Lumbar vertebrae	345	1.4505	23.8259
9	Cervical vertebrae/surgery	284	1.1940	25.0200
10	Lumbar vertebrae/pathology	278	1.1688	26.1888
11	Spinal stenosis/etiology	268	1.1268	27.3155
12	Laminectomy/methods	247	1.0385	28.3540
13	Spondylolisthesis/surgery	240	1.0090	29.3630
14	Intervertebral disc displacement/surgery	240	1.0090	30.3721
15	Spinal stenosis/pathology	237	0.9964	31.3685
16	Spinal stenosis/therapy	232	0.9754	32.3439
17	Spinal stenosis/physiopathology	214	0.8997	33.2436
18	Lumbar vertebrae/diagnostic imaging	210	0.8829	34.1266
19	Magnetic resonance Imaging	181	0.7610	34.8875
20	Decompression, Surgical	170	0.7147	35.6023
21	Magnetic resonance imaging/methods	159	0.6685	36.2708
22	Tomography, X-Ray computed	150	0.6306	36.9014
23	Spinal stenosis/drug therapy	138	0.5802	37.4816
24	Spinal fusion/instrumentation	135	0.5676	38.0492
25	Spinal fusion	134	0.5634	38.6126
26	Spinal fusion/adverse effects	127	0.5339	39.1465
27	Spinal cord compression/surgery	125	0.5255	39.6721
28	Laminectomy	120	0.5045	40.1766
29	Decompression, surgical/adverse effects	116	0.4877	40.6643
30	Spinal diseases/surgery	109	0.4583	41.1226
31	Cervical vertebrae/pathology	107	0.4499	41.5724
32	Spinal stenosis/epidemiology	105	0.4415	42.0139
33	Minimally invasive surgical procedures/methods	101	0.4246	42.4385
34	Cervical vertebrae/diagnostic imaging	96	0.4036	42.8421
35	Lumbar vertebrae/physiopathology	94	0.3952	43.2373
36	Cervical vertebrae	86	0.3616	43.5989
37	Postoperative complications/etiology	84	0.3532	43.9521
38	Intervertebral disc degeneration/surgery	80	0.3363	44.2884
39	Spinal stenosis/veterinary	78	0.3279	44.6164
40	Spinal cord compression/etiology	77	0.3237	44.9401
41	Laminectomy/adverse effects	77	0.3237	45.2638
42	Low back pain/surgery	76	0.3195	45.5834
43	Prostheses and implants	72	0.3027	45.8861
44	Thoracic vertebrae/surgery	68	0.2859	46.1720
45	Ligamentum flavum/pathology	66	0.2775	46.4494
46	Intervertebral disc displacement/diagnosis	63	0.2649	46.7143
47	Spinal diseases/diagnosis	62	0.2607	46.9750
48	Spondylosis/surgery	61	0.2565	47.2314
49	Quality of life	61	0.2565	47.4879
50	Spinal stenosis/rehabilitation	59	0.2481	47.7360
51	Spinal canal/surgery	59	0.2481	47.9840
52	Internal fixators	58	0.2439	48.2279
53	Decompression, surgical/instrumentation	58	0.2439	48.4717
54	Spinal osteophytosis/surgery	57	0.2396	48.7114
55	Spinal canal/pathology	56	0.2354	48.9468
56	Lumbar vertebrae/injuries	56	0.2354	49.1823
57	Low back pain/etiology	56	0.2354	49.4177

**Table 6:** High-frequency major MeSH a terms-source articles matrix (localized).

**No**.	**Major MeSH terms/MeSH subheadings**	**Pubmed Unique Identifiers of source articles**				
		**10,024,119**	**10,025,024**	**10,025,689**	**…**	**9,988,954**
1	Spinal stenosis/surgery	1	1	0	…	0
2	Lumbar vertebrae/surgery	1	0	0	…	0
3	Spinal stenosis/diagnosis	0	0	0	…	0
4	Decompression, surgical/methods	0	0	0	…	0
…	…	…	…	…	…	…
56	Lumbar vertebrae/injuries	0	0	0	…	0
57	Low back pain/etiology	0	0	0	…	0

## Discussion

We have examined previous literature concerning spinal stenosis using retrieval, characterization, and clustering. The number of articles published has generally maintained an upward trend with a narrow fluctuation. In 2011, an obvious decline occurred but the number rose again in the subsequent year. MeSH terms can reflect the main idea of the literature and the combination with a mass of accurate MeSH terms can reveal the hotspots and trends in research fields. Next, based on the statistical analysis of BICOMB software, “spinal stenosis” has always maintained the highest occurring frequency, despite declining in individual years with the main trend of articles. Finally, the software gCLUTO divided these articles into some clusters according to the similarity and co-word analysis of MeSH terms. Using the above process, we can identify the knowledge hierarchy and research anticipation of spinal stenosis.

Cluster 0 focuses on the effect of medical imaging on the diagnosis. In the final analysis, spinal stenosis is a pathological change caused by morphological changes of the spine column. Fluoroscopy examination is indispensable to determine the site, severity, and nature of the lesion. To characterize stenosis more precisely, studies have made detailed classifications according to different criteria in morphology. Stenosis development is closely associated with the anatomic variation, including that associated with vertebrae, soft tissue structure, spinal canal, and segmental sagittal diameter. Among them, the common choice to classify is the change in the spinal diameter at different segments and sections (13). The evolutionary tendency of stenosis, whether causing neuropathy and requiring surgical intervention or not, will be speculated according to the numerical standards based on morphologic change. Additionally, the narrowing degree in one segment may suggest corresponding changes and developing trends in other segments (14). Presently, clinicians manually segment the spinal canal area using CT images and measure the anteroposterior diameter of the spinal canal to diagnose whether the patient has spinal stenosis, leading to a workload and stronger subjectivity. The development of artificial intelligence contributes to precise mastery of morphologic imaging changes in disease progression by using deep learning technology in computer diagnosis.

Cluster 1 focuses on the etiology of spinal stenosis using different pathological symptoms. Spinal stenosis is only a generalization of the narrowing of the spinal canal in morphology. Various symptoms may be secondary to the physiological structure change and great differences exist in the causes of these concomitant symptoms. Several neurological symptoms, such as limb numbness, low back pain, and claudication, occur in the patients, most often due to the complication of nerve compression. Cervical stenosis usually results in limb paresthesia, dexterity dysfunction, imbalance, and neck pain (15). However, cervical spondylosis relates to lesion sites, tissue involvement degree, and individual differences and is classified into radiculopathy, myelopathy, and vertebral artery types instead of a simple disc-protrusion or slipped disc (16). Lumbar stenosis always leads to numbness in the lower limbs, low back pain, and even cauda equina compressive symptoms. According to the urgency of incidence, different pathogenic possibilities exist, such as laminar thickening, facet hyperplasia, and vertebral dislocation. Accurate analysis of the etiology is an inevitable requirement for further disposition in all circumstances.

Cluster 2 focuses on the pathogenesis of spinal stenosis including pathology structures, segments, and sections. Additionally, these mainly involve the ligamentum flavum and intervertebral disc, cervical and lumbar spine, and sagittal and transverse diameters. Morphological changes in the soft tissue around the spinal canal are the main characteristics of spinal stenosis. Ligamentum flavum forms the dorsal wall of the spinal canal, obviously causing narrowness when thickening. Regarding the reason for thickening, studies have identified the epiligament in the ligamentum flavum, comprising collagenous fibers, while enlargement of the epiligament led to more collagenous than natural elastic fibers and canal tapering. Furthermore, the ligamentum flavum area has been compared with more sensitive measurement parameters than the ligament flavum thickness, playing a more valuable role (17). Close to the ventral wall of the canal, the herniation of intervertebral disc would narrow the spinal canal. Similarly, at the ventral wall, ossification of the posterior longitudinal ligament is also a risk factor. Genetic mutations causing ectopic osteogenesis of the ligament may account for the ossification phenomenon (18). Until now, Pavlov's ratio, the ratio of the sagittal diameter of spinal canal to that of the corresponding vertebral body, is a reliable diagnostic basis of stenosis. However, tissues that are not bone structure shown on X ray are less studied. Magnetic Resonance Imaging (MRI), which reveals the pathological changes in soft tissues, is an appropriately effective auxiliary to clarify the pathogenesis. In recent studies, a comprehensive comparison was made between the Pavlov's ratio and MRI scan, revealing a moderate correlation (19). More specific and extensive evaluation of MRI images and traditional diagnostic criteria is conducive to dissect the complex pathogenesis and symptomatic treatment. Additionally, we believe molecular biology will promote the determination of pathogenesis through genomics, transcriptomics, and proteomics analysis of the ligaments and disc tissues. Targeted specific drug design is the research trend for stenosis treatment.

Cluster 3 focuses on the surgical method options under particular conditions and inspection of adverse effects in failure cases. According to the follow-up and postoperative recovery analysis of many past cases, it is not the optimal procedure for idiopathic symptoms. For instance, anterior decompression with spinal fusion occurred before treating cervical stenosis caused by posterior longitudinal ligament ossification (20), and laminectomy could improve radiculopathy effectively using foraminotomy (21). Doctors will formulate the most suitable surgical plan after discussing the patients' pathogenesis and evaluation of the operation. However, unpredictable postoperative symptoms and complications may persist following adequate treatment. Both standard open laminectomy and minimally invasive laminectomy are applied widely to treat cervical spondylosis using lamina trapping and ligament ossification (22). However, postoperative epidural fibrosis with a scar would restrict nerve mobility and increase tension, causing nerve injury and pain (23). Adhesion of the dura mater, canal wall, and ligamentum flavum can also cause cerebrospinal fluid leakage if a tear occurs. Additionally, intraoperative spinal cord injury can lead to paraplegia and incomplete decompression, operative area infection, and spinal atrophy. Regarding incision, anterior surgery exhibits a higher recovery rate and surgical risk, and posterior surgery shows more neurological deterioration. To improve recovery and prevent complications, clinicians strive to optimize the operation and control soft tissue injury and infection. Additionally, the optimization of foraminoscope technology and the improvement in minimally invasive spinal instruments, such as drills and trephines under the microscope, will help improve the success rate of surgery and control the risk of postoperative infection.

Cluster 4 focuses on the role of instrumentations in the rehabilitation of patients with spinal stenosis after decompression. As observed in previous operative cases, pure spinal fusion or laminectomy for decompression results in vertebral instability and increased mobility. Thus, more clinical and fundamental research continues to design multiple internal implantations to stabilize injured vertebral structures. The applied effect of each implantation is adequately inspected. E.H.Kuner evaluated the ASIF internal fixator for the operation of spinal fractures. They observed that internal fixation could effectively reduce the remaining encroachment and remodel the spinal canal, contributing to improving the operative effect and reducing operative complications (24). Since then, various interspinous process decompression (IPD) devices have been tested and applied clinically. These devices would relieve the load on facet joints, restore the height of the intervertebral foramen, and maintain the stability of the spine. However, a follow-up survey also revealed the existing risks, including spinous process fracture, cerebrospinal fluid leakage, and low-back pain (25). At the moment, the indications for these devices require more preoperative examination, intraoperative observation, and postoperative monitoring. Additionally, the prevention methods for post-IPD complications warrant further study.

Cluster 5 focuses on the surgical indications for clinical manifestations. As discussed previously, operative intervention is not inevitable and doctors made optimal plans in which operation would occur for specific conditions. Low back pain, a type of civilized disease prevalent in modern society, occurring mainly in the fourth and fifth lumbar or fifth lumbar and the first sacral vertebrae, is an important indication. Almost eighty percent of adults have pain with osteomyelitis, fracture, and spinal stenosis (26). Operation will be considered after falling flat, with conservative treatment for low back pain usually caused by disc herniation, spondylolisthesis, and degenerative disc disease. Minimizing the surgical trauma is also a critical consideration of the surgeon. Thus, in most cases, minimally invasive interventional approaches are the first choice. Orthopedic minimally invasive techniques such as foraminal, interlaminar, and delta mirrors, are widely adopted to improve function and reduce the side effect of drugs (27). Combined with clinical experience, we propose the following three indications for surgery: the patient has unbearable pain and requires surgery when treated conservatively; the patient has sustained intermittent neurogenic claudication and lower extremity symptoms for more than 2–3 months without significant improvement after conservative treatment; or the patient has severe dysfunction, such as progressive weakness of the lower limbs or incontinence. Additionally, surgical intervention using appropriate conservative intervention may maximize the therapeutic effect.

Cluster 6 focuses on the non-operative therapeutic means of spinal stenosis. Conservative treatment is favored because of the lack of collateral damage to the organism. The choice of conservative treatment vs. operation depends on accurate mastery of the clinical examinations including paraspinal tissue morphology, the functional status, and symptoms. Three major strategies are used—oral painkillers, epidural injection, and physical therapy—on the patients in the early stage with moderate symptoms clinically. Among them, epidural corticosteroid injection was confirmed to reduce pain, although the reduction was by a small degree and non-sustained (28). Additionally, epidural injection was more cost-effective and safe. The role of physical therapy in relieving pain and restoring function was limited, but necessary exercise therapy contributed to rehabilitation and complication prevention. Only functional training is less effective than operation in most cases. Non-steroidal anti-inflammatory drugs (NSAIDs) are the most common drug therapy clinically. The pharmacological mechanism involves reducing nerve stimulation, preventing painful secreted substances, and lowering the pain threshold. Thus, NSAIDs cannot fundamentally solve the problem but may reduce pain perception and may lead to drug dependence. Therefore, drug therapy is invalid and must improve. Overall, non-operative therapy is suitable preoperatively for mild to moderate patients and is available in preoperative preparation and postoperative rehabilitation. Additionally, traditional Chinese medicine, massage, and acupuncture show significant improvement effects in mild patients in clinical practice. For patients with different degrees and symptoms, formulating different treatment plans with integrated Chinese and Western medicine may produce the best therapeutic effect, which is also a research hotspot for the future.

As mentioned above, we have described the research status and further trends of spinal stenosis over the last 20 years research. However, reflective research loopholes and limitations may influence the analysis results. First, all the data were obtained from a single database and not involved in most language countries. High-quality literature in other databases was ignored. Collaborative analysis of multiple databases will help to improve the universality of the study. Second, co-word retrieval based on MeSH terms is limited with occasionality. The number and selection of MeSH terms will improve the biclustering results. Third, the range of our information retrieval starts from the origination of spinal stenosis studies, which may be a superiority, but could also weaken the recent research trends. Combining more measurement standards will contribute to capturing more research hotspots in the future.

## Conclusion

Among each stage of spinal stenosis onset, we conclude the hotspots in pathogenesis and therapeutic research. Pathogenesis has always been the focus of disease research, helping to master and judge progression. The ligmentum flavum and posterior longitudinal ligmentum, which compose the anteroposterior wall of the spinal canal, have attracted increased attention recently. Pathological changes in the ligmentum components, including hypertrophy and ossification, may alter the local morphology, leading to the narrowing of the spinal canal. Furthermore, publishers care more about the selection of therapeutic means in terms of severity. After long-term follow-up surveys and randomized controlled trials, operations combined with proper functional rehabilitation training could maximize the life quality of patients. Additionally, the operative principle is more inclined to minimal invasion and internal implantation, which lowers the risk of infection and postoperative complications. Further refining the pathological classification by optimizing the surgical method and instrument properties will be an important future direction for spinal stenosis.

## Data Availability Statement

The original contributions presented in the study are included in the article/supplementary material, further inquiries can be directed to the corresponding author/s.

## Author Contributions

LT: conceptualization, project administration, and writing—review and editing. SZ: data curation and methodology. KY: formal analysis, validation, and writing—original draft. LP: funding acquisition and investigation. KW: resources and software. All authors read and approved the manuscript.

## Conflict of Interest

The authors declare that the research was conducted in the absence of any commercial or financial relationships that could be construed as a potential conflict of interest.

## Abbreviations

BICOMB: Bibliographic Item Co-Occurrence Matrix Builder
MeSH: Medical Subject Headings
WoS: Web of Science Core Collection
NCBI: National Center for Biotechnology Information
JCR: Journal Citation Reports
MRI: Magnetic Resonance Imaging
ASIF: anterior subcutaneous internal fixator
IPD: interspinous process decompression
NSAIDs: Nonsteroidal anti-inflammatory drugs.

## References

[1] Miyauchi A Sumida T Kaneko M Manabe H Adachi N. Morphology and clinical importance of epidural membrane and periradicular fibrous tissue in lumbar spinal stenosis. Eur Spine J. (2017) 26:382–8. 10.1007/s00586-016-4640-z 27272620

[2] Du JP Fan Y Hao DJ. Rare hereditary abnormal bone hyperplasia and ossification of the yellow ligament complicated by thoracic spinal stenosis. World Neurosurg. (2018) 115:99–100. 10.1016/j.wneu.2018.04.035 29660555

[3] Melancia JL Francisco AF Antunes JL. Spinal stenosis. Handb Clin Neurol. (2014) 119:541–9. 10.1016/B978-0-7020-4086-3.00035-7 24365318

[4] Ogink PT Teunis T van Wulfften Palthe O Sepucha K Bono CM Schwab JH. Variation in costs among surgeons for lumbar spinal stenosis. Spine J. (2018) 18:1584–91. 10.1016/j.spinee.2018.02.015 29496622

[5] Forsth P Olafsson G Carlsson T Frost A Borgstrom F Fritzell P. A randomized, controlled trial of fusion surgery for lumbar spinal stenosis. New Engl J Med. (2016) 374:1413–23. 10.1056/NEJMoa1513721 27074066

[6] Ghogawala Z Dziura J Butler WE Dai F Terrin N Magge SN. Laminectomy plus fusion versus laminectomy alone for lumbar spondylolisthesis. New Engl J Med. (2016) 374:1424–34. 10.1056/NEJMoa1508788 27074067

[7] Yao RQ Ren C Wang JN Wu GS Zhu XM Xia ZF. Publication trends of research on sepsis and host immune response during 1999-2019: a 20-year bibliometric analysis. Int J Biol Sci. (2020) 16:27–37. 10.7150/ijbs.37496 31892843 PMC6930382

[8] Ahmad P Asif JA Alam MK Slots J. A bibliometric analysis of Periodontology 2000. Periodontology 2000. (2020) 82:286–97. 10.1111/prd.12328 31850637

[9] Yang S Kim CY Hwang S Kim E Kim H Shim H. COEXPEDIA: exploring biomedical hypotheses via co-expressions associated with medical subject headings (MeSH). Nucleic Acids Res. (2017) 45:D389–96. 10.1093/nar/gkw868 27679477 PMC5210615

[10] Synnestvedt MB Chen C Holmes JH. CiteSpace II: visualization and knowledge discovery in bibliographic databases. In: AMIA. Annual Symposium Proceedings. AMIA Symposium. Philadelphia, PA (2005). p. 724–8. PMC1560567 16779135

[11] Lei C. Development of a Text Mining System Based on the Co-occurrence of Bibliographic Items in Literature Databases. Shenyang: New Technology of Library & Information Service (2008).

[12] Wei WJ Shi B Guan X Ma JY Wang YC Liu J. Mapping theme trends and knowledge structures for human neural stem cells: a quantitative and co-word biclustering analysis for the 2013-2018 period. Neural Regener Res. (2019) 14:1823–32. 10.4103/1673-5374.257535 31169201 PMC6585554

[13] Miyazaki M Takita C Yoshiiwa T Itonaga I Tsumura H. Morphological analysis of the cervical pedicles, lateral masses, and laminae in developmental canal stenosis. Spine. (2010) 35:E1381–5. 10.1097/BRS.0b013e3181e8958f 21030896

[14] van Eck CF Spina NT III Lee JY. A novel MRI classification system for congenital functional lumbar spinal stenosis predicts the risk for tandem cervical spinal stenosis. Eur Spine J. (2017) 26:368–73. 10.1007/s00586-016-4657-3 27323965

[15] Rowe PC Marden CL Heinlein S Edwards CC II. Improvement of severe myalgic encephalomyelitis/chronic fatigue syndrome symptoms following surgical treatment of cervical spinal stenosis. J Transl Med. (2018) 16:21. 10.1186/s12967-018-1397-7 29391028 PMC5796598

[16] Hohmann D Kugelgen B Liebig K. [Chronic spondylogenic cervical myelopathy. Pathogenesis Prognosis, Therapy. (1985) 14:101–11. 4000672

[17] Kim YU Park JY Kim DH Karm MH Lee JY Yoo JI. The role of the ligamentum flavum area as a morphological parameter of lumbar central spinal stenosis. Pain Phys. (2017) 20:E419–24. 10.36076/ppj.2017.E424 28339441

[18] Wang P Liu X Kong C Liu X Teng Z Ma Y. Potential role of the IL17RC gene in the thoracic ossification of the posterior longitudinal ligament. Int J Mol Med. (2019) 43:2005–14. 10.3892/ijmm.2019.4130 30864693 PMC6443333

[19] Prasad SS O'Malley M Caplan M Shackleford IM Pydisetty RK. MRI measurements of the cervical spine and their correlation to Pavlov's ratio. Spine. (2003) 28:1263–8. 10.1097/01.BRS.0000065570.20888.AA 12811269

[20] Masaki Y Yamazaki M Okawa A Aramomi M Hashimoto M Koda M. An analysis of factors causing poor surgical outcome in patients with cervical myelopathy due to ossification of the posterior longitudinal ligament: anterior decompression with spinal fusion versus laminoplasty. J Spinal Disord Tech. (2007) 20:7–13. 10.1097/01.bsd.0000211260.28497.35 17285045

[21] Snow RB Weiner H. Cervical laminectomy and foraminotomy as surgical treatment of cervical spondylosis: a follow-up study with analysis of failures. J Spinal Disord. (1993) 6:245–50; discussion 250–1. 10.1097/00002517-199306030-00011 8347976

[22] Alimi M Hofstetter CP Pyo SY Paulo D Hartl R. Minimally invasive laminectomy for lumbar spinal stenosis in patients with and without preoperative spondylolisthesis: clinical outcome and reoperation rates. J Neurosurg Spine. (2015) 22:339–52. 10.3171/2014.11.SPINE13597 25635635

[23] Lin CL Jou IM Wu CY Kuo YR Yang SC Lee JS. Topically applied cross-linked hyaluronan attenuates the formation of spinal epidural fibrosis in a swine model of laminectomy. Sci Rep. (2019) 9:14613. 10.1038/s41598-019-50882-x 31601849 PMC6787060

[24] Kuner EH Schlickewei W Kuner A Hauser U. Restoration of the spinal canal by the internal fixator and remodeling. Eur Spine J. (1997) 6:417–22. 10.1007/BF01834072 9455672 PMC3467722

[25] Gazzeri R Galarza M Neroni M Fiore C Faiola A Puzzilli F. Failure rates and complications of interspinous process decompression devices: a European multicenter study. Neurosurg Focus. (2015) 39:E14. 10.3171/2015.7.FOCUS15244 26424338

[26] van den Berg R Jongbloed EM de Schepper EIT Bierma-Zeinstra SMA Koes BW Luijsterburg PAJ. The association between pro-inflammatory biomarkers and nonspecific low back pain: a systematic review. Spine J. (2018) 18:2140–51. 10.1016/j.spinee.2018.06.349 29960111

[27] Urits I Burshtein A Sharma M Testa L Gold PA Orhurhu V. Low back pain, a comprehensive review: pathophysiology, diagnosis, and treatment. Curr Pain Headache Rep. (2019) 23:23. 10.1007/s11916-019-0757-1 30854609

[28] Chou R Hashimoto R Friedly J Fu R Bougatsos C Dana T. Epidural corticosteroid injections for radiculopathy and spinal stenosis: a systematic review and meta-analysis. Ann Int Med. (2015) 163:373–81. 10.7326/M15-0934 26302454

